# PSMC5 insufficiency and P320R mutation impair proteasome function

**DOI:** 10.1093/hmg/ddae085

**Published:** 2024-05-22

**Authors:** Zhong-Qiu Yu, Jenny Carmichael, Galen A Collins, Maria Daniela D'Agostino, Mathieu Lessard, Helen V Firth, Pooja Harijan, Andrew E Fry, John Dean, Jiuchun Zhang, Usha Kini, Alfred L Goldberg, David C Rubinsztein

**Affiliations:** Cambridge Institute for Medical Research, The Keith Peters Building, Department of Medical Genetics, University of Cambridge, Cambridge Biomedical Campus, Hills Road, Cambridge CB2 0XY, United Kingdom; UK Dementia Research Institute, University of Cambridge, The Keith Peters Building, Cambridge Biomedical Campus, Hills Road, Cambridge CB2 0XY, United Kingdom; Department of Clinical Genetics, Cambridge University Hospitals NHS Foundation Trust, Box 134, Addenbrooke’s Hospital, Cambridge CB2 0QQ, United Kingdom; Department of Cell Biology, Harvard Medical School, 240 Longwood Avenue, Boston, MA 02115, United States; Department of Biochemistry, Molecular Biology, Entomology, and Plant Pathology, Mississippi State University, 32 Creelman Street, Starkville MS 39762, United States; Division of Medical Genetics, Department of Specialised Medicine, McGill University Health Centre, 1001 Decarie Boulevard, Montreal, Quebec, H4A 3J1, Canada; Care for Rare Canada Consortium, Children’s Hospital of Eastern Ontario Research Institute, 401 Smyth Road, Ottawa, K1H 8L1, ON, Canada; Division of Medical Genetics, Department of Specialised Medicine, McGill University Health Centre, 1001 Decarie Boulevard, Montreal, Quebec, H4A 3J1, Canada; Care for Rare Canada Consortium, Children’s Hospital of Eastern Ontario Research Institute, 401 Smyth Road, Ottawa, K1H 8L1, ON, Canada; Department of Clinical Genetics, Cambridge University Hospitals NHS Foundation Trust, Box 134, Addenbrooke’s Hospital, Cambridge CB2 0QQ, United Kingdom; Department of Paediatric Neurosciences, Box 107, Child development centre, Addenbrooke’s Hospital, Hills Road, Cambridge CB2 0QQ, United Kingdom; All Wales Medical Genomics Service, University Hospital of Wales, Heath Park, Cardiff CF14 4XW, United Kingdom; Division of Cancer and Genetics, Cardiff University, Heath Park, Cardiff CF14 4XN, United Kingdom; The School of Medicine, Medical Sciences and Nutrition, Polwarth Building, University of Aberdeen, Aberdeen, AB25 2ZD, United Kingdom; Department of Cell Biology, Harvard Medical School, 240 Longwood Avenue, Boston, MA 02115, United States; Oxford Centre for Genomic Medicine, Oxford University Hospitals NHS Foundation Trust, Oxford & Radcliffe Department of Medicine, University of Oxford, Windmill Road, Oxford, OX3 7HE, United Kingdom; Department of Cell Biology, Harvard Medical School, 240 Longwood Avenue, Boston, MA 02115, United States; Cambridge Institute for Medical Research, The Keith Peters Building, Department of Medical Genetics, University of Cambridge, Cambridge Biomedical Campus, Hills Road, Cambridge CB2 0XY, United Kingdom; UK Dementia Research Institute, University of Cambridge, The Keith Peters Building, Cambridge Biomedical Campus, Hills Road, Cambridge CB2 0XY, United Kingdom

**Keywords:** proteasome, developmental delay, PSMC5, protein degradation, neurodevelopmental disorder

## Abstract

The ubiquitin-proteasome system mediates the degradation of a wide variety of proteins. Proteasome dysfunction is associated with neurodegenerative diseases and neurodevelopmental disorders in humans. Here we identified mutations in PSMC5, an AAA ATPase subunit of the proteasome 19S regulatory particle, in individuals with neurodevelopmental disorders, which were initially considered as variants of unknown significance. We have now found heterozygotes with the following mutations: P320R (6 individuals), R325W, Q160A, and one nonsense mutation at Q69. We focused on understanding the functional consequence of PSMC5 insufficiency and the P320R mutation in cells and found that both impair proteasome function and activate apoptosis. Interestingly, the P320R mutation impairs proteasome function by weakening the association between the 19S regulatory particle and the 20S core particle. Our study supports that proteasome dysfunction is the pathogenic cause of neurodevelopmental disorders in individuals carrying *PSMC5* variants.

## Graphical Abstract

**Graphical Abstract:**
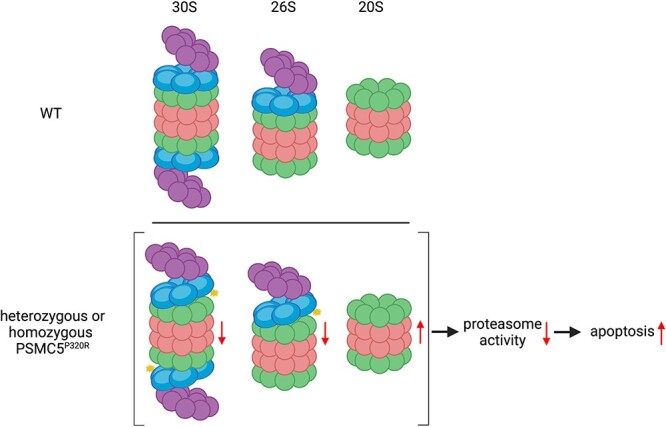

## Introduction

The ubiquitin-proteasome system (UPS) is a crucial protein degradation pathway that eliminates short-lived regulatory proteins and misfolded or damaged proteins in eukaryotic cells. Substrate proteins are typically first covalently attached to ubiquitin through the concerted action of an E1 ubiquitin-activating enzyme, an E2 ubiquitin-conjugating enzyme, and an E3 ubiquitin ligase. The ubiquitination serves as a recognition signal enabling the substrate to be degraded by the 26S proteasome [1, 2]. The 26S proteasome consists of the 20S core particle (CP), which hydrolyzes the unfolded substrate proteins into short peptides, and the 19S regulatory particle (RP), which recognizes the ubiquitin signal, deubiquitylates and unfolds substrate proteins, opens the gated channel of the 20S CP, and aligns the substrate translocation channel of 19S RP with the open gate of 20S CP [3–5]. The 20S CP has a barrel-shaped structure formed by four stacked hetero-heptameric rings of α(1-7)β(1-7)β(1-7)α(1-7), with β1, β2, and β5 possessing caspase-like, trypsin-like, and chymotrypsin-like proteolytic activities, respectively [4, 5]. As a relatively dynamic and heterogeneous subcomplex, the 19S RP roughly comprises 19 subunits containing six AAA ATPase subunits (Rpt1-6 in yeast and PSMC1-6 in human) and 13 non-ATPase subunits (Rpns in yeast and PSMDs in human) [6]. Typically, the 19S RP binds to one or both ends of the 20S CP to form proteasome holoenzymes, which are accordingly termed single-capped 26S proteasomes and double-capped 30S proteasomes [4, 7]. In addition, a stand-alone uncapped 20S CP, also called 20S proteasome, can directly mediate proteolysis of intrinsically disordered proteins or peptides [8, 9].

The UPS plays indispensable roles in neuronal function and development [10–13]. As the final destination for degradation of UPS substrates, the proteasome is crucial in maintaining the health of the human nervous system. Proteasome dysfunction has been implicated in the pathogenesis of neurodegenerative diseases including Alzheimer’s disease [14], Parkinson’s disease [15], Huntington’s disease [16], and amyotrophic lateral sclerosis [17]. Furthermore, mutations in genes encoding proteasome subunits result in neurodevelopmental disorders, as exemplified by variants of genes encoding the 20S CP subunit PSMB1 (β1) [18] and the 19S RP subunits PSMC3 [19, 20] and PSMD12 [21–23].

In this study, we initially ascertained two unrelated individuals both carrying the identical *de novo* P320R mutation in *PSMC5* presenting with neurodevelopmental delay and intellectual disability. This was initially considered as a variant of unknown significance. To clarify the biological significance and impact on neurodevelopment of *PSMC5* dysfunction and especially of its P320R variant, we ascertained more individuals harbouring *PSMC5* variants and conducted biochemical studies.

## Results

### Identification of *de novo PSMC5* variants—Case reports

The first *de novo PSMC5* missense variant (c.959C>G p.P320R) was detected in two patients (Cases 1 and 2), both showing moderate to severe developmental delay, speech delay, motor delay, and intellectual disability (Table 1). Later, 4 additional individuals, labelled Cases 3 and 4 and 2 further cases in the publicly available DECIPHER database were found to carry the same missense P320R variant, suggesting that this is a mutation hotspot. Parallel clinical work identified three other individuals with similar phenotypes carrying distinct *PSMC5* variants, including Case 5 carrying a nonsense mutation at Q69, Case 6 harbouring a missense mutation of Q160A, and Case 7 with a missense mutation of R325W. The *PSMC5* variants in Cases 6 & 7 (detailed in this study) and two additional cases listed in the DECIPHER database were identified by the Deciphering Developmental Disorders (DDD) study. All cases were associated with *de novo* mutations except for Case 6, where we have no evidence that this mutation occurred *de novo* or was inherited.

**Table 1:** Summary clinical features of individuals with PSMC5 mutations described in this paper.

	Case 1	Case 2	Case 3	Case 4	Case 5	Case 6	Case 7
Genetic Variant (NM_002805.6)	c.959C>G p.P320R	c.959C>G p.P320R	c.959C>G p.P320R	c.959C>G p.P320R	c.205C>T p.Q69Ter	c.479A>C p.Q160A	c.973C>T p.R325W
Current age	14 yrs	9 yrs	5 yrs	21 yrs	5 yrs	24 yrs	23 yrs
Sex	Male	Male	Female	Male	Female	Female	Male
Pregnancy and birth (weeks of gestation)	40 + 5/40, pain and poly-hydramnios	41/40, polyhydramnios	40/40, mild bilateral hydronephrosis	Term	37/40	41 + 3	39/40
Birth weight	3.18 kg	3.22 kg	3.085 kg	2.8 kg	2.67 kg	3.685 kg	Not available
NEURODEVELOPMENT							
Developmental Delay	YES	YES—severe	YES	YES	YES	YES—mild	YES—moderate
Speech delay	YES—non-verbal until the age of 4 yrs	YES—severe	YES—severe, non-verbal at 24 months	YES	YES	YES	YES
Motor delay	YES	YES—moderate	YES—moderate	YES—walked at 20 months	YES	YES—mild and persistently unsteady gait	YES
Intellectual disability	YES—moderate	YES—severe	YES	YES—severe	YES—moderate	YES—dyspraxia, dyslexia, borderline ID (1^st^ to 2^nd^ centile in several areas at 15 yrs)	YES—moderate
Regression	NO	YES	NO	NO	NO	NO	NO
OTHER NEUROLOGY							
Epilepsy/Seizures	NO	NO	NO	NO	NO	NO	Single nocturnal seizure at 11 yrs
MRI abnormalities	NO	YES—thin corpus callosum	YES—mild unilateral cerebellar heterotopia	Mild cortical atrophy	YES—hypomyelination	Not done	No information
Micro/Macrocephaly	NO	NO	NO	NO	NO	NO	NO
Sleep disturbances	NO	YES	NO	YES	YES, on melatonin	YES—restless	NO
Abnormal movements	NO	YES	NO	NO	NO	NO	NO
Hypotonia	YES	YES	YES	NO	YES	NO	NO
Vision	YES –moderate myopia, surgery for bilateral strabismus	YES	No information	NO	YES (moderate optic nerve dysfunction/atrophy and squint	Normal vision	Strabismus
Hearing impairment	NO	YES	NO	NO	NO	NO	YES—recurrent left otitis media, and ear wax
Facial dysmorphology	YES—prominent forehead	YES—narrow palpebral fissures, left eye ptosis, short philtrum with thin upper lip	YES—triangular face, prominent forehead, pointed eyebrows and mild synophrys, low nasal bridge and a short upturned nose, small but normally placed ears. There are prominent forehead veins and thin, translucent skin	YES—prominent forehead, narrow palpebral fissures, protruding columella, flared nares, wide mouth, thin lips	NO	NO	YES—prominent forehead, narrow palpebral fissures, prominent columnella, thin upper lip, multiple lentigines
BEHAVIOUR							
ASD/Autistic features	YES	YES	NO	YES, Asperger	YES	YES, Asperger syndrome diagnosed at 5 yrs	NO
ADD/ADHD	YES	YES	NO	NO	YES	NO but difficulty concentrating and often restless	NO
Anxiety	YES	NO	No information	YES	NO	YES	NO
Obsessive behaviour	YES	YES	No information	NO	YES	YES	No information
Emotional lability	YES	NO	No information	YES	YES	YES	NO
Aggressive behaviour	YES	YES	No information	YES	YES	YES	NO
GENERAL HEALTH							
Haematological abnormalities (e.g anaemia, thrombocytopaenia)	NO	NO	NO	NO	No information	NO	NO
Gastrointestinal symptoms (e.g reflux, feeding problems, constipation)	NO	No information	NO	Difficulty with chewing/swallowing food	YES—constipation	YES—constipation (occasional)	Imperforate anus, constipation
Joint hypermobility	NO	NO	YES	NO	NO	YES	NO
Current Height	No information	No information	3^rd^ centile	165 cm	2^nd^ centile	163.5 cm (+0.17 SD, 15 yrs)	145.1 cm
Current Weight	No information	No information	No information	73 kg	9^th^ centile	75.5 kg (+2.13 SD, 15 yrs)	75.6 kg
Current Head circumference	No information	No information	50^th^ centile	Unavailable	6^th^ centile	53.7 cm (−1.06 SD, 15 yrs)	13^th^ centile
Other ocular abnormalities (strabismus)	YES—surgery for strabismus	YES—bilateral optic nerve hypoplasia	No information	NO	YES	NO	Strabismus
Other musculoskeletal features (e.g. contractures, scoliosis)	YES—trigger finger on the right hand	NO	NO	NO	NO	Positional talipes, simple congenital torticollis at birth, mild right-sided plagiocephaly	Brachydactyly, tapering fingers, broad halluces
Cardiac anomalies	Rhythm abnormalities	Rhythm abnormalities	NO	NO	NO	NO	No Information
Dental features	NO	YES—pointy teeth	NO	NO	NO	YES—malocclusion with crowding in the right upper quadrant	No information

#### Case 1 (P320R)

This is a 14-year-old boy, the second child of healthy unrelated Caucasian parents. There is no other significant family history. He was born at 40 + 5 weeks of gestation following induction of labour with a birth weight of 3.18 kg. The pregnancy was complicated by pain and polyhydramnios. He was born with a contracture of his right middle finger, and he had generalised hypotonia during his infancy and subsequently developed some stiffness. His development was significantly delayed. He sat unsupported at 9 months, commando crawled at 9–10 months and walked independently at 20 months. His speech was significantly delayed and he was non-verbal until the age of 4–5 years. He required surgery for bilateral squint and wears glasses for significant myopia. He has significant problems with impulsive and at times aggressive behaviour and was diagnosed with autism and ADHD. He attends a special needs school and is on ADHD medication. There is no history of regression or epilepsy. He has been diagnosed with rhythm abnormalities on ECG.

#### Case 2 (P320R)

This is a 9-year-old boy, the second child of unrelated parents and there is no family history of significance. The pregnancy was complicated by polyhydramnios and concerns about ventriculomegaly. He was born at 41 weeks gestation with a birth weight of 3.22 kg. He presented with unilateral left sided ptosis after birth and was diagnosed with bilateral optic nerve hypoplasia. His development was significantly delayed across all areas and his MRI showed a thin corpus callosum. He has moderate to severe global developmental delay and intellectual disability. There is no history of regression or epilepsy. He has significant behavioural problems with autism, ADHD, obsessive and aggressive behaviour. He has hypotonia, vision and hearing impairment. He has some distinctive facial features with narrow palpebral fissures, left ptosis, short philtrum and a thin upper lip and a broad nasal tip. An ECHO showed an abnormally shaped aorta.

#### Case 3 (P320R)

This is a 5-year-old girl born to healthy unrelated parents, following assisted conception (ICSI) for decreased sperm motility. The pregnancy was uncomplicated. Mild bilateral hydronephrosis was diagnosed on the second trimester anomaly scan. She was born by spontaneous vaginal delivery at term with a birth weight 3.085 kg in good condition. The birth was complicated by meconium aspiration, otherwise there were no immediate neonatal concerns; the child was followed since birth for mild bilateral hydronephrosis without renal function compromise (currently in resolution) and hemangioma of the right shoulder which resolved spontaneously.

Hypotonia was noted from 5 months of age, with significant torticollis and plagiocephaly, for which positional and helmet treatment were required for 4 months. Fine and gross motor delay were also noted from 5 months of age. At 36 months of age the child had hypotonia and generalized joint hypermobility, severe and persistent gross motor delay, global developmental delay, severe expressive language delay (non-verbal at 24 months) and moderate receptive language delay. On clinical examination the child has short stature with height at 3^rd^ percentile, head circumference at the 50^th^ percentile and some distinctive facial features with a triangular face, prominent forehead, pointed eyebrows and mild synophorus, low nasal bridge and a short, upturned nose, and small but normally placed ears. There are prominent forehead veins and thin, translucent skin.

An MRI scan has shown a 3 mm nodular heterotopia of grey matter in the cerebellar hemisphere and no other structural or myelination abnormalities.

#### Case 4 (P320R)

This 21-year-old man is the second child of unrelated healthy parents. He was born following a normal pregnancy and delivery. He was noted to be dysmorphic from birth with a prominent forehead. Initial concerns were raised due to his delayed development. He started walking at 20 months of age. His speech was significantly delayed with his first words coming at 3 years. He can now talk in long sentences, but the clarity of his speech remains poor. He attended a Special School and is currently in college learning daily living skills.

He has been diagnosed with autism spectrum disorder. He is reported to show “islands of intelligence”, for example he knows the flags of all countries. His behaviour can be challenging with him becoming aggressive with frustration.

He has a distinctive facial appearance with a broad forehead, narrow palpebral fissures, protruding columella, flared nares, short philtrum, and wide mouth with thin lips.

Brain imaging at 18 months of age is reported to show cerebral atrophy.

Additionally, 2 further cases with very similar clinical profile of neurodevelopmental delay and speech and language delay with the PSMC5 P320R mutation are visible in DECIPHER [24].

#### Case 5 (Q69Ter)

This is the first child of non-consanguineous parents. There is no significant family history. She was born at 37 weeks after induction of labour by normal vaginal delivery with a birth weight of 2.67 kg. The early months of her life were uneventful. She presented at 4–5 months with episodic horizontal nystagmus and poor visual attention. The motor milestones were delayed—she sat independently at 9 months, crawled just before 2 years of age and walked independently after her second birthday. She had generalised hypotonia with normal EMG and nerve conduction studies. Her speech has been significantly delayed and at the age of 5 years she has single words. She does not point and does not use gestures to communicate. There is no history of regression or epilepsy. At the age of 5 years she displays significant autistic traits, ADHD and aggressive behaviour. There are no facial dysmorphic features and her growth parameters are between the 2–9^th^ centiles. There are no reported dental problems. She has moderate intellectual disability and attends a special needs school with 1:1 support. Due to the abnormal eye movements and developmental delay the girl had an MRI scan at the age of 2 years, which showed significant hypomyelination of the cerebral and cerebellar white matter and she was diagnosed with moderate optic nerve dysfunction and a squint. There are no other medical problems but does suffer with significant constipation for which she requires regular medication and night terrors and is on melatonin for sleep disturbance.

#### Case 6 (Q160A)—DECIPHER 304265

This 24-year-old female is the first child of unrelated parents. The patient was born at 41 + 3 weeks gestation following an uncomplicated pregnancy with a birth weight of 3.68 kg. She was born with positional talipes and congenital torticollis. She had mild motor developmental delay, walking independently at 16 months but her gait remained persistently unsteady. When assessed at 3 years of age she was not dysmorphic but was noted to have a mild right sided plagiocephaly, poor spatial awareness, speech delay and coordination problems. Assessment at 15 years of age indicated her intellectual abilities were 1st to 2nd centile in several areas. At 24 years of age, she has mild intellectual disability, dyslexia, dyspraxia and joint hypermobility. She has no facial dysmorphic features but does have small hands. The patient experiences difficulty concentrating and significant anxiety which causes restlessness at night. She is poor at following verbal instructions but has no hearing or vision problems. She continues to have poor coordination. The patient is known to carry a maternally inherited balanced paracentric inversion of chromosome 4 (q31.3q34.1q35.1). Array CGH was normal.

#### Case 7 (R325W)—DECIPHER 261007

This is a 23-year-old man. He was born following an uncomplicated pregnancy. He had an imperforate anus at birth (not detected on scans at 20 weeks) which was treated surgically. His main subsequent problems have been failure to thrive, short stature and intellectual disability. He had a single nocturnal seizure at the age of 11 years and there is no history of behavioural problems or autistic behaviour. He had strabismus and distinctive facial features, which include a prominent forehead, narrow palpebral fissures, prominent columella, thin upper lip and multiple lentigines. Skeletal features of note are brachydactyly, tapering fingers and broad halluces.

Of note, all the *PSMC5* variants mentioned above have arisen *de novo* and are heterozygous (except for Case 6 where we have no evidence that this was *de novo* or inherited). Thus, it seems possible that defective or deficient PSMC5 contributes to the pathogenesis of neurodevelopmental disorders. To investigate this hypothesis, we focused on the functional influence of PSMC5 depletion and PSMC5 P320R mutation in cells in further studies of this work.

### PSMC5 depletion increases the accumulation of K48-ubiquitinated proteins and activates apoptosis

**Figure 1:**
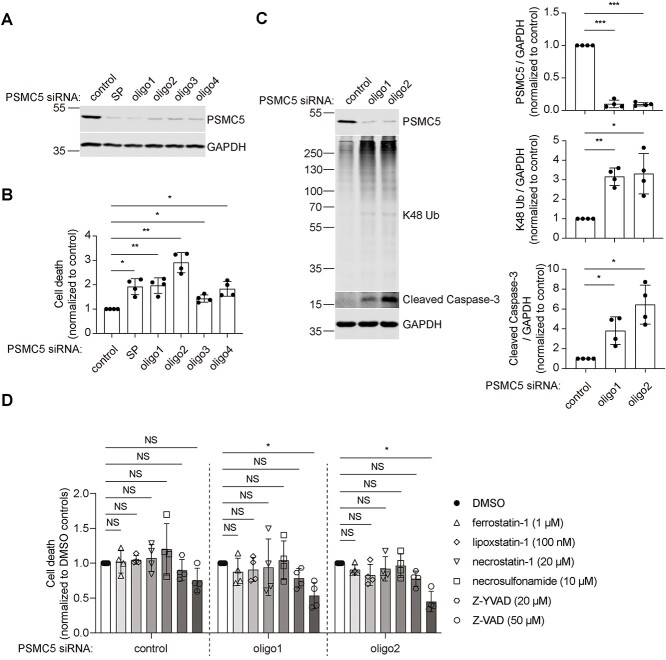
PSMC5 knockdown results in accumulation of K48-ubiquitinated proteins and apoptotic cell death in HeLa cells. (A) Western blotting of PSMC5 levels after PSMC5 siRNA knockdown using SMARTpool or four deconvoluted oligos. GAPDH served as protein loading control. (B) LDH cytotoxicity assay to measure cell death caused by PSMC5 knockdown. Plots represent mean ± SD (n = 4 independent experiments). *P* values were calculated using two-tailed, paired Student’s t-test. (C) Western blotting and quantification of PSMC5, K48-ubiquitinated proteins, and cleaved caspase-3 levels in PSMC5-knockdown cells. GAPDH served as protein loading control. Plots represent mean ± SD (n = 4 independent experiments). *P* values were calculated using two-tailed, paired Student’s t-test. (D) LDH cytotoxicity assay to measure the inhibition effect of ferroptosis inhibitors (ferrostatin-1, liproxstatin-1), necroptosis inhibitors (necrostatin-1, necrosulfonamide), pyroptosis inhibitor (Z-YVAD), and apoptosis inhibitor (Z-VAD) on cell death caused by PSMC5 knockdown. Cell death of drug treatment groups was normalized to DMSO treated wild-type, PSMC5 knockdown with oligo1 or oligo2, respectively. Plots represent mean ± SD (n = 4 independent experiments). P values were calculated using one-way ANOVA with post-hoc Dunnett’s multiple comparison test. ^*^ indicates *P* < 0.05; ^*^^*^ indicates *P* < 0.01; ^*^^*^^*^ indicates *P* < 0.001; NS, not significant.

PSMC5 was knocked down in human cervical cancer HeLa cells and human neuroblastoma SH-SY5Y cells. We first tested the efficiency of PSMC5 knockdown using SMARTpool small interfering RNA (siRNA) and four distinct single oligonucleotides (deconvoluted from the pool in the SMARTpool) in HeLa cells (Fig. 1A). All the siRNA-mediated knockdown of PSMC5 increased cell death over 48 h (Fig. 1B). We chose oligo1 and oligo2 to knock down PSMC5 in SH-SY5Y cells, and both worked efficiently and increased cell death (Fig. S1A–C). Hereafter, oligo1 and oligo2 were used for further dissecting the influence of PSMC5 depletion on cells. PSMC5 is an essential subunit of the 19S RP that recognizes, deubiquitylates, and unfolds substrates so that they can enter the 20S proteolytic CP and be degraded into short peptides or amino acids. Lysine-48 (K48) ubiquitinated proteins chains are well established as substrates for proteasomal degradation, therefore we next examined whether depleting PSMC5 affects the accumulation of K48-ubiquitinated proteins [25]. As expected, HeLa and SH-SY5Y cells with PSMC5 knockdown exhibited enhanced accumulation of K48-ubiquitinated proteins (Fig. 1C and Fig. S1C), suggesting that the function of proteasomes is impaired in when PSMC5 is knocked down.

As proteasome inhibitors induce apoptosis in several cell lines [26–28], we investigated whether apoptosis is induced in PSMC5-knockdown HeLa and SH-SY5Y cells. PSMC5 knockdown in HeLa and SH-SY5Y cells induced the cleavage of caspase-3 (Fig. 1C and Fig. S1C), an executioner in apoptosis, suggesting that PSMC5 depletion activates apoptosis. Consistent with the role of apoptosis in the PSMC5 knockdown-induced cell death, PSMC5-knockdown cells treated with apoptosis inhibitor Z-VAD (50 μM), but not ferroptosis inhibitors ferrostatin-1 (1 μM) and liproxstatin-1 (100 nM), necroptosis inhibitors necrostatin-1 (20 μM) and necrosulfonamide (10 μM), or pyroptosis inhibitor Z-YVAD (20 μM), were rescued from cell death (Fig. 1D and Fig. S1D). Taken together, these data indicate that depletion of PSMC5 impairs the function of proteasome and activates apoptosis.

### Depletion of 19S subunits impairs proteasome function and activates apoptosis

**Figure 2:**
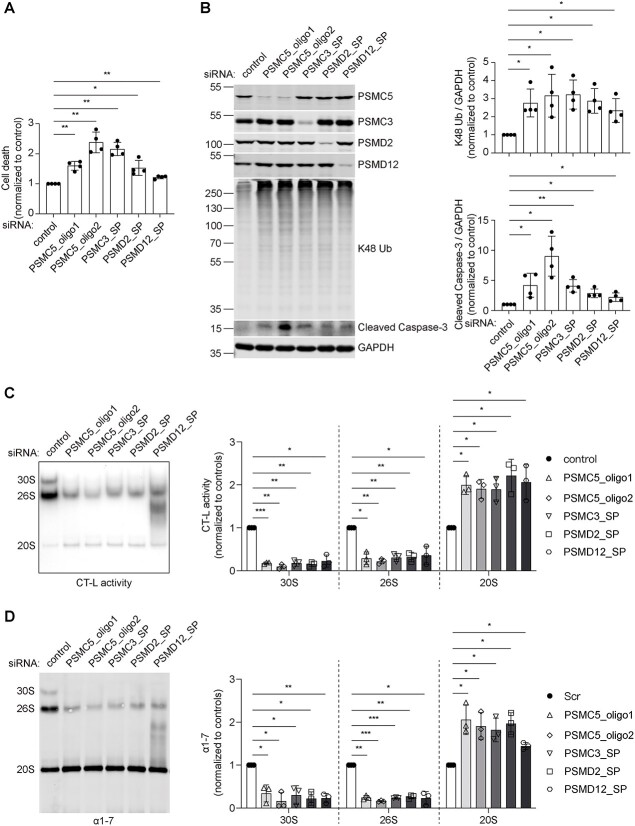
Knockdowns of the 19S RP subunits of the 26S proteasome impairs proteasome function and leads to apoptosis in HeLa cells. (A) LDH cytotoxicity assay to measure cell death caused by PSMC5, PSMC3, PSMD2, or PSMD12 siRNA knockdown. Plots represent mean ± SD (n = 4 independent experiments). *P* values were calculated using two-tailed, paired Student’s t-test. (B) Western blotting of PSMC5, PSMC3, PSMD2, PSMD12, K48-ubiquitinated proteins, and cleaved caspase-3 levels, and quantification of K48-ubiquitinated proteins and cleaved caspase-3 in PSMC5, PSMC3, PSMD2, or PSMD12-knockdown cells. GAPDH served as protein loading control. Plots represent mean ± SD (n = 4 independent experiments). *P* values were calculated using two-tailed, paired Student’s t-test. (C) In-gel proteasome chymotrypsin-like (CT-L) activity of the 30S, 26S, and 20S proteasome complexes and quantification thereof in PSMC5, PSMC3, PSMD2, or PSMD12-knockdown cells. The CT-L activity of proteasome complexes is visualized by their ability to cleave the Suc-LLVY-AMC fluorogenic peptide. The CT-L activity of the 30S, 26S, and 20S proteasome complexes of different knockdowns was normalized to the corresponding controls in the quantification plots. Plots represent mean ± SD (n = 3 independent experiments). *P* values were calculated using two-tailed, paired Student’s t-test. (D) Immunoblotting of the native gel in (C) and probing of the membrane with anti-α1-7 antibody and quantification the amount of 30S, 26S, and 20S proteasome complexes indicated by α1-7. The protein level of α1-7 in the 30S, 26S, and 20S proteasome complexes of different knockdowns was normalized to the corresponding controls in the quantification plots. Plots represent mean ± SD (n = 3 independent experiments). *P* values were calculated using two-tailed, paired Student’s t-test. ^*^ indicates *P* < 0.05; ^*^^*^ indicates *P* < 0.01; ^*^^*^^*^ indicates *P* < 0.001; NS, not significant.

Along with P320R mutation in PSMC5, pathogenic truncated or missense variants of other subunits of the 19S RP: AAA ATPase subunit PSMC3 [19, 20] and non-ATPase subunits PSMD2 [29] and PSMD12 [21–23, 30], were reported in recent years. We next investigated whether depletion of other subunits of the 19S RP has effects similar to PSMC5 depletion. siRNA mediated knockdowns of PSMC3, PSMD2, and PSMD12 in HeLa cells recapitulated the phenotypes of PSMC5 knockdown, including increased cell death, enhanced accumulation of K48-ubiquitinated proteins, and cleavage of caspase-3 (Fig. 2A and B, and Fig. S2A). We observed a tendency for the depletion of a specific subunit of the 19S RP to upregulate the protein levels of other subunits of the 19S RP and subunits of the 20S CP (Fig. 2B and Fig. S2). This may be explained by the Nrf1-mediated transcriptional upregulation of the proteasome during proteasome impairment to compensate for reduced proteasome function [31].

To further address how the depletion of subunits of the 19S RP impairs proteasome function, we performed an in-gel proteasome assay to quantify the activity and composition of different proteasome complexes using total cell lysates [32]. Using a fluorogenic short peptide substrate (Suc-LLVY-AMC), cells from siRNA knockdown control exhibited proteasome chymotrypsin-like (CT-L) activity at the bands of double-capped 30S proteasome, single-capped 26S proteasome, and uncapped 20S proteasome (Fig. 2C). Knockdowns of PSMC5, PSMC3, PSMD2, and PSMD12 significantly diminished the CT-L activity of 30S proteasomes and 26S proteasomes but enhanced the CT-L hydrolysis by 20S proteasomes. Subsequent immunoblotting of the native gel used in the in-gel proteasome assay with anti-α1-7 antibody (Fig. 2D), as well as anti-β5, anti-PSMC5, anti-PSMC3, anti-PSMD2, anti-PSMD12 antibodies (Fig. S3), suggested that the diminished 30S proteasome and 26S proteasome activity observed in PSMC5, PSMC3, PSMD2, and PSMD12-knockdown cells was associated with a decreased pool of 30S proteasome complexes and 26S proteasome complexes. Likewise, the enhanced 20S activity in PSMC5, PSMC3, PSMD2, and PSMD12-knockdown cells was associated with an increased pool of 20S proteasome complexes revealed by native gel immunoblotting with anti-α1-7 and anti-β5 antibodies (Fig. 2D and Fig. S3). In addition, MG132 treatment obviously diminished the bands of 30S, 26S, and 20S proteasomes in the in-gel proteasome activity assay while keeping the bands of 30S, 26S, and 20S proteasomes unaffected in immunoblotting of the same native gel with anti-α1-7 and anti-PSMC3 antibodies (Fig. S4), confirming that the in-gel proteasome assay using Suc-LLVY-AMC as a substrate validly reflects proteasome activity. These data indicate that the depletion of subunits of the 19S RP impairs proteasome function by disassembling double-capped 30S proteasome and single-capped 26S proteasome into uncapped 20S proteasome.

### Transiently expressing PSMC5^P320R^ in PSMC5-knockdown cells impairs the association of the 19S RP with the 20S CP

**Figure 3:**
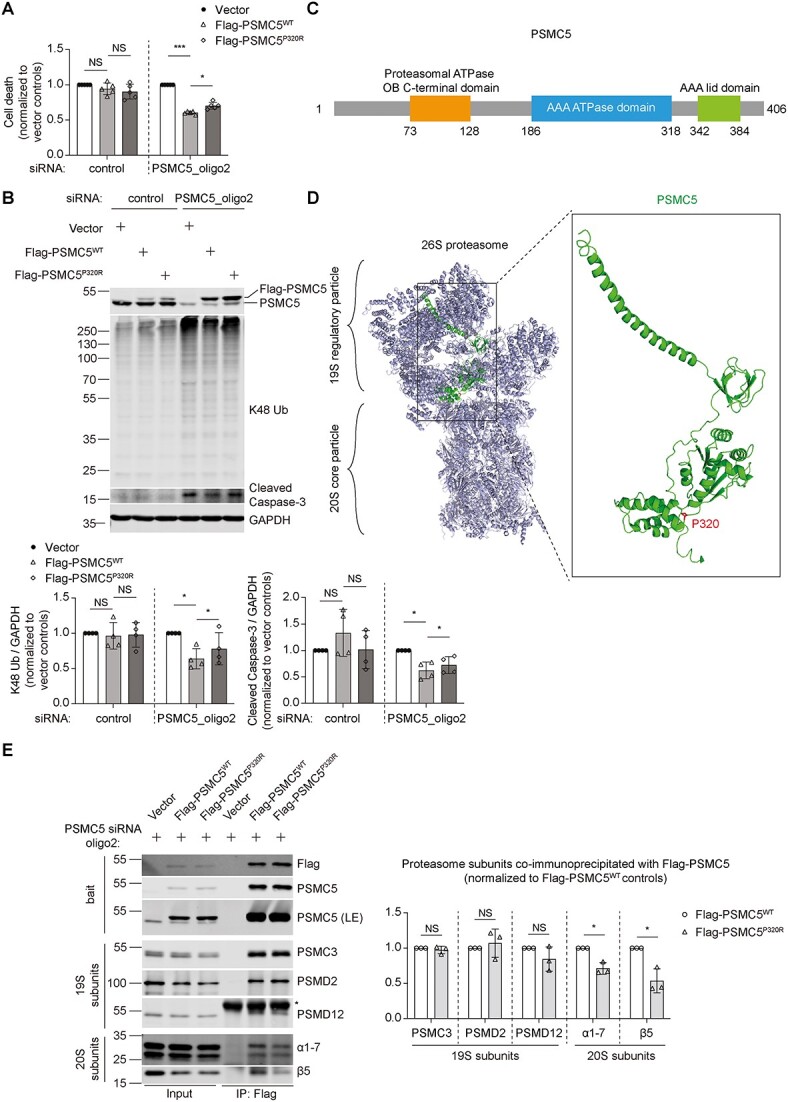
Transient expression of PSMC5^P320R^ in PSMC5-knockdown HeLa cells leads to the dissociation of the 19S RP from the 20S CP. (A) LDH cytotoxicity assay to compare cell death in PSMC5-knockdown HeLa cells transiently expressing Flag-PSMC5^WT^ or PSMC5^P320R^. Cell death of control and PSMC5 knockdown groups was normalized to the corresponding vector groups, respectively. Plots represent mean ± SD (n = 5 independent experiments). *P* values were calculated using two-tailed, paired Student’s t-test. (B) Western blotting of PSMC5, K48-ubiquitinated proteins, and cleaved caspase-3 levels and quantification of K48-ubiquitinated proteins, and cleaved caspase-3 in PSMC5-knockdown HeLa cells transiently expressing Flag-PSMC5^WT^ or PSMC5^P320R^. GAPDH served as protein loading control. The protein levels of K48-ubiquitinated proteins and cleaved caspase-3 were normalized to the corresponding vector groups in the quantification plots. Plots represent mean ± SD (n = 4 independent experiments). *P* values were calculated using two-tailed, paired Student’s t-test. (C) Domain organization of PSMC5. (D) the structure of 26S proteasome and PSMC5 are shown as ribbon diagrams with PSMC5 highlighted in green and PSMC5^P320^ highlighted in red. The ribbon diagrams were generated using PyMOL software based on the solved structure of the human 26S proteasome (PDB 6MSH) [34]. (E) Immunoprecipitation of exogenously expressed Flag-PSMC5^WT^ or PSMC5^P320R^ in PSMC5-knockdown HeLa cells and quantification of the 19S RP and 20S CP subunits co-immunoprecipitated with Flag-PSMC5^WT^ or PSMC5^P320R^. PSMC5 (LE) indicates a longer exposure of the PSMC5 blot. ^*^ in the blots indicates non-specific bands. The amount of all the preys (PSMC3, PSMD2, PSMD12, α1-7, and β5) co-immunoprecipitated with Flag-PSMC5 was normalized to the corresponding bait Flag-PSMC5 and then normalized to the corresponding Flag-PSMC5^WT^ groups in the quantification plots. Plots represent mean ± SD (n = 3 independent experiments). *P* values were calculated using two-tailed, paired Student’s t-test. ^*^ indicates *P* < 0.05; ^*^^*^ indicates *P* < 0.01; NS, not significant. Endogenous PSMC5 is knocked down using PSMC5 siRNA oligo2 and exogenously expressed PSMC5^WT^ or PSMC5^P320R^ are PSMC5 siRNA oligo2-resistant.

To dissect the pathogenic mechanisms of PSMC5 P320R mutation, we next transiently expressed Flag-PSMC5^P320R^ or its wild-type counterpart in PSMC5-knockdown HeLa cells. Both constructs ameliorated the cell death, caspase-3 cleavage, and the accumulation of K48-ubiquitinated proteins seen in PSMC5-knockdown cells—however, the rescue of these consequences was attenuated by the P320R mutation (Fig. 3A and B). These data suggest that PSMC5 P320R mutation impairs proteasome function, which may make cells more prone to apoptosis. We noted that in the control cells the upper Flag-tagged PSMC5 bands were weaker in the wild-type cells than in the PSMC5 knockdown cells (Fig. 3B). This is likely because the tagged proteins are incorporated into proteasomes more effectively in the knockdown cells where they do not need to compete with the untagged endogenous protein. Since the PSMC5 that is not incorporated into proteasomes will be unstable [33], this likely explains these observations.

P320 in PSMC5 is located outside of the AAA ATPase domain (Fig. 3C) and at the interface of the 19S RP and the 20S CP [34] (Fig. 3D). We hypothesized that mutation of this residue may impede the association of the 19S RP and the 20S CP. We performed co-immunoprecipitation experiments using Flag-PSMC5^WT^ or Flag-PSMC5^P320R^ as bait to test this hypothesis. We found that the amount of subunits of the 19S RP (PSMC3, PSMD2, and PSMD12) co-immunoprecipitated with Flag-PSMC5^P320R^ remained unchanged compared to immunoprecipitation with wild-type PSMC5 (Fig. 3E). However, the amount of subunits of the 20S CP (α1-7 and β5) co-immunoprecipitated with Flag-PSMC5^P320R^ was decreased compared to wild-type PSMC5 (Fig. 3E). These data suggest that the PSMC5 P320R mutation does not affect the integrity of the 19S RP complexes but impairs the association of the 19S RP with the 20S CP.

### Both homozygous and heterozygous PSMC5^P320R^ activate apoptosis and impair proteasome function by dissociating the 19S RP from the 20S CP

**Figure 4:**
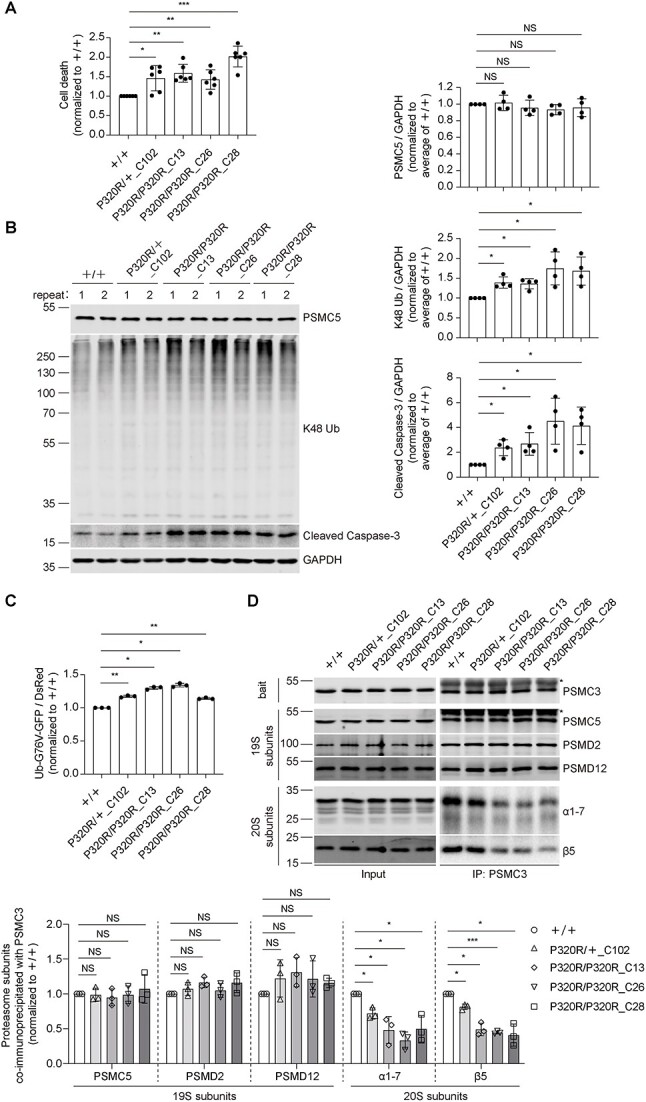
Engineered BE(2)-M17 cells carrying homozygous or heterozygous PSMC5^P320R^ show accumulation of K48-ubiquitinated proteins, apoptosis, and the dissociation of the 19S RP from the 20S CP. (A) CellTox green cytotoxicity assay to measure cell death of BE(2)-M17 cells carrying homozygous or heterozygous PSMC5^P320R^. Plots represent mean ± SD (n = 6 independent experiments). *P* values were calculated using two-tailed, paired Student’s t-test. (B) Western blotting and quantification of PSMC5, K48-ubiquitinated proteins, and cleaved caspase-3 levels in BE(2)-M17 cells carrying homozygous or heterozygous PSMC5^P320R^. GAPDH served as protein loading control. The average of two repeats shown in one western blot image was taken as a biological replicate. Plots represent mean ± SD (n = 4 independent experiments). *P* values were calculated using two-tailed, paired Student’s t-test. (C) BE(2)-M17 cells carrying homozygous or heterozygous PSMC5^P320R^ and wild-type control were transfected with Ub-G76V-GFP-IRES-DsRed, followed by FACS. Plots represent mean ± SD (n = 3 independent experiments). *P* values were calculated using two-tailed, paired Student’s t-test. (D) Immunoprecipitation of PSMC3 in BE(2)-M17 cells carrying homozygous or heterozygous PSMC5^P320R^ and quantification of the 19S RP and 20S CP subunits co-immunoprecipitated with PSMC3. ^*^ in the blots indicates non-specific bands. The amount of all the preys (PSMC5, PSMD2, PSMD12, α1-7, and β5) co-immunoprecipitated with PSMC3 was normalized to the corresponding PSMC3 and then normalized to the corresponding +/+ groups in the quantification plots. Plots represent mean ± SD (n = 3 independent experiments). *P* values were calculated using two-tailed, paired Student’s t-test. ^*^ indicates *P* < 0.05; ^*^^*^ indicates *P* < 0.01; ^*^^*^^*^ indicates *P* < 0.001; NS, not significant. +/+: Wild type; P320R/+: Heterozygous PSMC5^P320R^; P320R/P320R: Homozygous PSMC5^P320R^. C13, clone 13; C26, clone 26; C28, clone 28; C102, clone 102.

To mimic the pathogenic effect of PSMC5 P320R mutation in patients on a homogeneous genetic background, we generated one neuroblastoma BE(2)-M17 cell line carrying heterozygous PSMC5^P320R^, as well as three BE(2)-M17 cell lines carrying homozygous PSMC5^P320R^. Compared with the wild-type BE(2)-M17 (+/+), both homozygous (P320R/P320R: C13, clone 13; C26, clone 26; C28, clone 28) and heterozygous PSMC5^P320R^ (P320R/+: C102, clone 102) cells exhibited increased cell death and cleavage of caspase-3 (Fig. 4A and B). The increased cell death caused by PSMC5 P320R mutation in heterozygous and homozygous PSMC5^P320R^ cell lines were significantly inhibited by treatment with the apoptosis inhibitor Z-VAD (50 μM) (Fig. S5A), confirming that the increased cell death in these cell lines is predominantly apoptotic.

The enhanced accumulation of K48-ubiquitinated proteins in homozygous and heterozygous PSMC5^P320R^ lines suggest that proteasome function is compromised (Fig. 4B). To further confirm the impaired proteasome function in homozygous and heterozygous PSMC5^P320R^ cells, we transfected BE(2)-M17 cell lines with Ub-G76V-GFP-IRES-DsRed, which enables co-expression of Ub-G76V-GFP and free DsRed from a single CMV promoter in one expression construct. The 26S proteasome rapidly degrades Ub-G76V-GFP, but free DsRed is comparatively resistant to the degradation by proteasomes. Thus, the ratio of the fluorescence intensity of GFP to DsRed of each cell in FACS can be used as a quantitative indicator of proteasome activity. As expected, the degradation of Ub-G76V-GFP in homozygous and heterozygous PSMC5^P320R^ was significantly inhibited (Fig. 4C), indicating that proteasome activity is impaired. Further, co-immunoprecipitation experiments using the 19S RP subunit PSMC3 as bait revealed that the 19S RP complexes remain intact, but the association of the 19S RP with the 20S CP is impaired in cells expressing PSMC5^P320R^ (Fig. 4D).

We note that PSMC5 P320R mutation did not obviously affect the protein levels of subunits of the 19S RP (PSMC5, PSMC3, PSMD2, and PSMD12) and slightly upregulated the protein levels of subunits of the 20S CP (α1-7 and β5) (Fig. 4B and Fig. S5B), excluding the possibility that the activated apoptosis and impaired proteasome function in PSMC5^P320R^-expressing cells are due to lower levels of proteasome subunits.

### PSMC5^P320R^ disassembles 30S proteasome (and 26S proteasome) into 20S proteasome

**Figure 5:**
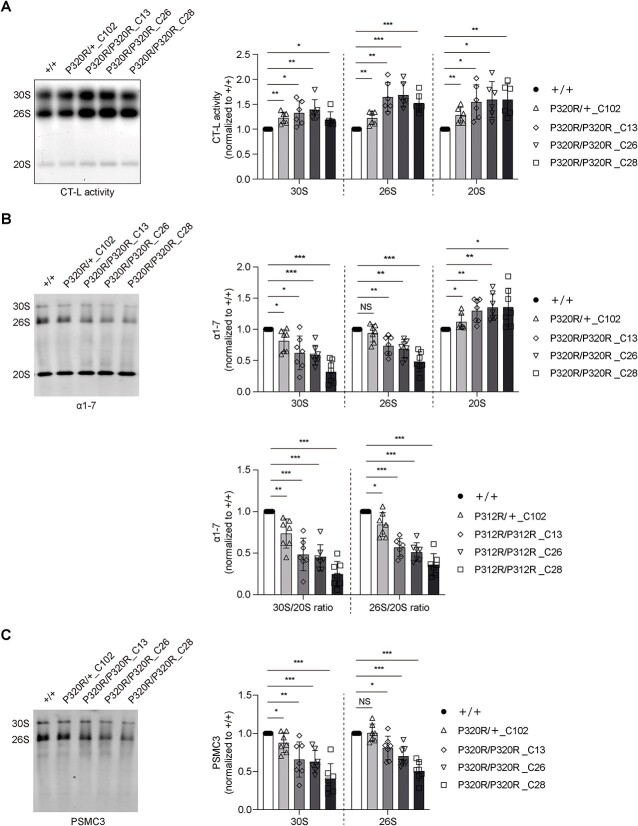
Homozygous PSMC5^P320R^ shows the decrease of 26S and 30S proteasome and the increase of 20S proteasome, and heterozygous PSMC5^P320R^ shows the decrease of 30S proteasome and the increase of 20S proteasome. (A) Proteasome CT-L activity in BE(2)-M17 cells carrying homozygous or heterozygous PSMC5^P320R^ was assessed in gels using the Suc-LLVY-AMC fluorogenic peptide and quantification thereof. The CT-L activity of the 30S, 26S, and 20S proteasome complexes in different cell lines was normalized to the corresponding +/+ groups in the quantification plots. Plots represent mean ± SD (n = 6 independent experiments). P values were calculated using two-tailed, paired Student’s t-test. (B) Immunoblotting of the native gel in (A) and probing of the membrane with anti-α1-7 antibody and quantification the amount of 30S, 26S, and 20S proteasome complexes or the ratio of 30S/20S and 26S/20S indicated by α1-7. The protein level of α1-7 in the 30S, 26S, and 20S proteasome complexes or in the ratio of 30S/20S and 26S/20S in different cell lines was normalized to the corresponding +/+ groups in the quantification plots. Plots represent mean ± SD (n = 7 independent experiments). *P* values were calculated using two-tailed, paired Student’s t-test. (C) Immunoblotting of the native gel in (A) and probing of the membrane with anti-PSMC3 antibody and quantification the amount of 30S and 26S proteasome complexes indicated by PSMC3. The protein level of PSMC3 in the 30S, 26S, and 20S proteasome complexes in different cell lines was normalized to the corresponding +/+ groups in the quantification plots. Plots represent mean ± SD (n = 7 independent experiments). *P* values were calculated using two-tailed, paired Student’s t-test. ^*^ indicates *P* < 0.05; ^*^^*^ indicates *P* < 0.01; ^*^^*^^*^ indicates *P* < 0.001; NS, not significant. +/+: Wild type; P320R/+: Heterozygous PSMC5^P320R^; P320R/P320R: Homozygous PSMC5^P320R^. C13, clone 13; C26, clone 26; C28, clone 28; C102, clone 102.

Counterintuitively, when performing an in-gel proteasome assay to quantify the activity and different proteasome complexes in homozygous and heterozygous PSMC5^P320R^ BE(2)-M17 cell lines, we found that CT-L activity of 30S proteasome, 26S proteasome, and 20S proteasome all increased (Fig. 5A). However, immunoblotting of native gels with anti-α1-7 antibody and anti-PSMC3 antibody showed that homozygous PSMC5^P320R^ cells had decreased amounts of 26S and 30S proteasome and slightly increased 20S proteasome, and heterozygous PSMC5^P320R^ cells had decreased 30S proteasome and slightly increased 20S proteasome (Fig. 5B and C). Considering (1) the impaired proteasome function shown by increased accumulation of K48-ubiquitinated proteins (Fig. 4B) and decreased degradation of Ub-G76V-GFP (Fig. 4C) and (2) decreased amount of 30S proteasome and 26S proteasome (or unchanged amount of 26S proteasome) (Fig. 5B and C) in homozygous and heterozygous PSMC5^P320R^ BE(2)-M17 cells, the reason for increased in-gel 30S and 26S proteasome CT-L activity in homozygous and heterozygous PSMC5^P320R^ cells is presumably that besides dissociating the 19S RP from the 20S CP, PSMC5 P320R mutation also opens a crack between the 19S RP and the 20S CP in intact 30S and 26S proteasomes. This crack allows the entry of short peptide substrate Suc-LLVY-AMC, but not ubiquitinated proteins, into the 20S CP.

## Discussion

In this study, we identified three heterozygous PSMC5 missense variants: P320R, R325W, and Q160A, and one nonsense variant: Q69, from cases with neurodevelopmental disorders, and discovered that insufficiency of PSMC5 (and also other subunits of the 19S RP) and the PSMC5 P320R mutation impair proteasome function and activate apoptosis. Specifically, the PSMC5 P320R mutation weakens the association between the 19S RP and the 20S CP, while keeping the 19S RP intact. This decreases the amount of 30S and 26S proteasome. This inherently impairs proteasome function. The homozygous cells have generally greater impairment of proteasome activity (Fig. 4C) but there is some variation between lines, and more K48 ubiquitin-linked proteins (Fig. 4B). In the homozygotes, all 26S and 30S proteasomes will be affected. In heterozygotes, only 50% of 26S will be affected, but maybe 75% of 30S proteasome will be affected (because they will have either 0, 1 or 2 mutant PSMC5 subunits). Thus, the differences in 30S proteasome function may not be that obvious when comparing the homozygote and heterozygote cell lines (Fig. 5A).

This mutation may impair proteasome proteasome function in three other ways. (1) PSMC5 incorporation is one of the last steps in assembling the 19S ATPase ring [35] and requires the binding of the chaperone PSMD10/gankyrin at its C-terminal end [36] near where the P320R mutation sits. Thus, we cannot rule out an assembly defect on top of the destabilizing effects of the mutation. (Issues with the N-terminal chaperone for PSMC5, PAAF1, likely leads to neurodegeneration in mice [33].) Interestingly, the p.Ala324_Lys360del mutation in PSMC5 has been reported to affect its incorporation into the 26S proteasome, although the impact of this mutation from a clinical perspective was difficult to assess as the individual harbors mutations in two other proteasome subunits [37]. (2) Although PSMC5 lacks a HbYX motif and thus does not directly open the 20S CP gated channel [7, 38], the P320R mutation may hamper the gate opening of the 20S CP by loosening the connection of HbYX motif-containing AAA ATPase subunits PSMC1, PSMC3, and PSMC4 with the 20S CP; (3) PSMC5 P320R mutation opens a crack between the 19S RP and the 20S CP of the remaining intact 30S and 26S proteasome, and this may cause difficulty of aligning the substrate translocation channel of the 19S RP with the open gate of the 20S CP. This will likely lead to the impaired degradation of ubiquitinated substrates that we observed, even in the context of our observation that the 20S proteasome in P320R homozygous cells showed no defect in the ability to degrade artificial peptide substrates that do not require ubiquitination. Interestingly, this model would predict that immunoproteasomes (which have different beta subunits in the 20S) may also have the same defect as we have observed and it would be interesting to test if this is the case in future work.

As proteasome defects lead to compensatory responses like activation of autophagy, the integrated stress response, and Nrf1 transcriptional programmes [31, 39, 40], it is possible that some of the consequences of these proteasome mutations may be driven by hyperactive compensatory programmes.

Previous published work has shown that nonsense or missense variants of proteasome subunits PSMB1 [18], PSMC3 [19, 20], and PSMD12 [22, 23] are associated with neurodevelopmental disorders. These variants affect the protein levels of proteasome subunits, or decreases their incorporation into 20S and 26S proteasome and also the assembly of 20S and 26S proteasome [18–20, 22, 23]. The results of the functional studies presented in this work, as well as those of prior studies demonstrating a link between disruption of proteasome subunit function and neurodevelopmental disorders, strongly support an association between haploinsufficiency of *PSMC5* and the neurodevelopmental features observed in patients harboring *de novo* variants in this gene. Animal models would likely be necessary to further understand the exact mechanism by which variants in this gene result in the observed clinical features in humans. It will be interesting in future work to understand the developmental pathways that may be altered by proteasome compromise and assess whether there is an element of proteotoxicity that contributes to the phenotypes.

## Materials and methods

### Institutional review board statement

For the purposes of this study IRB review was waived and informed patient/guardian consent was given for publication of de-identified data and the appropriate institutional forms have been archived in accordance with the principles stated in the Declaration of Helsinki. Participants did not receive a stipend.

### Informed consent statement

The individuals reported were patients referred to the clinical genetics service for investigations of global developmental delay and intellectual disability and PSMC5 variants were identified via trio whole genome sequencing. All individuals had signed informed consent according to institutional review boards and local regulatory authorities for participating in diagnostic or research studies.

### Cell lines

Human cervical cancer HeLa cell line was cultured in Dulbecco’s modified Eagle’s medium (DMEM) (Sigma-Aldrich, D6546) supplemented with 10% Fetal Bovine Serum (FBS) (Sigma-Aldrich, F7524), 2 mM L-glutamine (Sigma-Aldrich, G7513), and 100 U/mL Penicillin–Streptomycin (Sigma-Aldrich, P0781), or in DMEM (Sigma-Aldrich, D6529) supplemented with 10% FBS (Sigma-Aldrich, F7524) and 100 U/mL Penicillin–Streptomycin (Sigma-Aldrich, P0781). Human neuroblastoma SH-SY5Y and BE(2)-M17 cell lines were cultured in DMEM/F12 (Sigma-Aldrich, D6421) supplemented with 10% Fetal Bovine Serum (FBS) (Sigma-Aldrich, F7524), 2 mM L-glutamine (Sigma-Aldrich, G7513), 1× MEM non-essential amino acid (Sigma-Aldrich, M7145), and 100 U/mL Penicillin–Streptomycin (Sigma-Aldrich, P0781), or in DMEM/F12 (Sigma-Aldrich, D8437) supplemented with 10% Fetal Bovine Serum (FBS) (Sigma-Aldrich, F7524), 1× MEM non-essential amino acid (Sigma-Aldrich, M7145), and 100 U/mL Penicillin–Streptomycin (Sigma-Aldrich, P0781). All cell lines were maintained in 5% CO_2_ at 37°C and routinely tested for mycoplasma contamination.

To generate BE(2)-M17 cells harboring heterozygous and homozygous PSMC5^P320R^, the transfected cells (generated by Synthego) were sorted as single cells into 96-well plates by flow cytometry. Mutants were identified by Illumina MiSeq with primers 5′-TCTGCTCAGGTTATCATGGCTA and 5′-GCAATTTTTCTCAGGTTGATCC.

### Antibodies and reagents

The following antibodies have been used in this work (Table 2): rabbit anti-PSMC5 (Abcam, ab178681), mouse anti-GAPDH (Proteintech, 60004-1), rabbit anti-GAPDH (Cell Signaling, 2118L), rabbit anti-K48-linkage specific polyubiquitin (Cell signaling, 8081S), rabbit anti-cleaved caspase-3 antibody (Cell signaling, 9661S), rabbit anti-caspase-3 antibody (Cell signaling, 9662S), rabbit anti-PSMC3 (Proteintech, 24142-1-AP), rabbit anti-PSMD2 (Proteintech, 14748-1-AP), mouse anti-PSMD12 (Santa Cruz, sc-398279), mouse anti-20S proteasome α1 + 2 + 3 + 5 + 6 + 7 (Abcam, ab22674), mouse anti-20S proteasome α1 + 2 + 3 + 5 + 6 + 7 (Merck, ST1049), mouse anti-20S proteasome β5 (Santa Cruz, sc-393931), mouse anti-FLAG (Sigma-Aldrich, F1804), donkey anti-rabbit IgG, horseradish peroxidase (HRP)-conjugated secondary antibody (Cytiva, NA934V), goat anti-rabbit IgG H&L, (HRP)-conjugated secondary antibody (Abcam, ab205718), goat anti-mouse IgG H&L, (HRP)-conjugated secondary antibody (Abcam, ab205719), goat anti-mouse IgG (H + L) secondary antibody DyLight™ 680 conjugated (Invitrogen, 35518), goat anti-rabbit IgG (H + L) secondary antibody DyLight™ 800 4× PEG conjugate (Invitrogen, SA535571), goat anti-rabbit IgG (H + L) secondary antibody DyLight™ 680 conjugated (Invitrogen, 35568), goat anti-mouse IgG (H + L) secondary antibody DyLight™ 800 4× PEG conjugate (Invitrogen, SA535521). All the primary antibodies were used at a dilution of 1:1000, except anti-GAPDH antibodies at a dilution of 1:5000, anti-β5 antibody at a dilution of 1:200, and anti-PSMD12 antibody at a dilution of 1:100. The HRP-conjugated secondary antibodies were used at a dilution of 1:2000. The DyLight fluorochromes-conjugated secondary antibodies were used at a dilution of 1:5000.

**Table 2:** Antibodies used in this paper.

ANTIBODIES	SOURCE	IDENTIFIER
Mouse anti-20S proteasome α1 + 2 + 3 + 5 + 6 + 7	Abcam	ab22674
Mouse anti-20S proteasome α1 + 2 + 3 + 5 + 6 + 7	Merck	ST1049
Mouse anti-20S proteasome β5	Santa Cruz	sc-393 931
Rabbit anti-caspase-3 antibody	Cell signaling	9662S
Rabbit anti-cleaved caspase-3 antibody	Cell signaling	9661S
Mouse anti-FLAG	Sigma-Aldrich	F1804
Mouse anti-GAPDH	Proteintech,	60004-1
Rabbit anti-GAPDH	Cell Signaling	2118L
Rabbit anti-K48-linkage specific polyubiquitin	Cell signaling	8081S
Rabbit anti-PSMC3	Proteintech	24142-1-AP
Rabbit anti-PSMC5	Abcam	ab178681
Rabbit anti-PSMD2	Proteintech	14748-1-AP
Mouse anti-PSMD12	Santa Cruz	sc-398 279
Donkey anti-rabbit IgG, (HRP)-conjugated secondary antibody	Cytiva	NA934V
Goat anti-rabbit IgG H&L, (HRP)-conjugated secondary antibody	Abcam	ab205718
Goat anti-mouse IgG H&L, (HRP)-conjugated secondary antibody	Abcam	ab205719
Goat anti-mouse IgG (H + L) secondary antibody DyLight™ 680 conjugated	Invitrogen	35518
Goat anti-rabbit IgG (H + L) secondary antibody DyLight™ 800 4× PEG conjugate	Invitrogen	SA535571
Goat anti-rabbit IgG (H + L) secondary antibody DyLight™ 680 conjugated	Invitrogen	35568
Goat anti-mouse IgG (H + L) secondary antibody DyLight™ 800 4× PEG conjugate	Invitrogen	SA535521

The following drugs have been used in this work: caspase inhibitor Z-VAD-FMK (Promega, G7231), caspase-1 inhibitor Z-YVAD-FMK (Abcam, ab141388), ferrostatin-1 (MedChemExpress, HY-100579), liproxstatin-1 (MedChemExpress, HY-12726), necrostatin-1 (Sigma-Aldrich, N9037), necrosulfonamide (Abcam, ab143839), proteasome inhibitor MG132 (Sigma-Aldrich, C2211). Cell death inhibitors were added to HeLa and SH-SY5Y cells 4 h after siRNA knockdown and incubated with cells for about two days. Cell death inhibitor treatments in BE(2)-M17 cells were performed after BE(2)-M17 cells attached to the bottom of the wells and incubated with cells for about two days. Proteasome inhibitor treatment in HeLa cells for in-gel proteasome assay was performed after HeLa cells were collected and lysed using OverKleeft (OK) lysis buffer.

### Plasmids and siRNAs

The following DNA constructs were used in this study: pcDNA3.1-PSMC5^WT^ (PSMC5 siRNA oligo2-resistant), pcDNA3.1-PSMC5^P320R^ (PSMC5 siRNA oligo2-resistant), and pUb-G76V-GFP-IRES-DsRed. The pcDNA3.1-PSMC5^WT^ (PSMC5 siRNA oligo2-resistant) and pcDNA3.1-PSMC5^P320R^ (PSMC5 siRNA oligo2-resistant) were constructed using QuikChange II XL Site-Directed Mutagenesis Kit (Agilent Technologies, 200521), following manufacturer’s instructions, using the following primer: 5′-GaaCgtGcaAtcGgtGtgatgagccacagcccgggccaacag-3′ and 5′-aCacCgaTtgCacGttCattcgtgtctctggctctgaactgg-3′. The pUb-G76V-GFP-IRES-DsRed was constructed by subcloning Ub-G76V-GFP from Ub-G76V-GFP plasmid (gift from Nico Dantuma, Addgene, plasmid #11941) [41] into pIRES2 DsRed-Express2 vector between EcoRI and BamHI restriction sites using Gibson assembly method (NEB, E2611S).

Pre-designed siRNAs (ON-TARGETplus SMARTpool and/or set of deconvoluted oligos) from GE Healthcare Dharmacon): control non-targeting siRNA (D-001810-10), PSMC5 SMARTpool (L-009484-00-0005), PSMC5 oligo1 (5′-CCAAGAACAUCAAGGUUAU-3′), PSMC5 oligo2 (5′-CAUACGGACUGUACCUUUA-3′), PSMC5 oligo3 (5′-CAAGGUAGACCCAUUAGUG-3′), PSMC5 oligo4 (5′-GGAACAUGCUCCAUCUAUC-3′), PSMC3 SMARTpool (L-008738-00-0005), PSMD2 SMARTpool (L-017212-00-0005), PSMD12 SMARTpool (L-011368-01-0005).

### Transfection

TransIT-2020 Transfection Reagent (Mirus, MIR5400) and GeneXplus Transfection Reagent (ATCC, ACS-4004) were used for DNA transfection, while Lipofectamine 2000 Transfection Reagent (Invitrogen, 11668019) and Lipofectamine RNAiMax Transfection Reagent (Invitrogen, 13778150) were used for siRNA transfection, according to the manufacturer’s instructions. For the siRNA knockdown experiments, HeLa cells and SH-SY5Y cells in suspension were transfected with 50 nM siRNA using Lipofectamine 2000 and Lipofectamine RNAiMax, respectively. Cells were harvested after two days of siRNA transfection.

For transient expression of PSMC5^WT^ or PSMC5^P320R^ in PSMC5-knockdown HeLa cells, cells in suspension were transfected with DNA using GeneXplus, at a DNA (μg) to GeneXplus (μL) ratio of 1:3. Cells were split 24 h after DNA transfection and further transfected with 25 nM siRNA using Lipofectamine 2000. Cells were harvested after three days of siRNA transfection. To summarise, on Day 1 cells were transfected with plasmids, on Day 2 cells were split and transfected with siRNAs, and on Day 5 cells were harvested.

For DNA transfection in BE(2)-M17 cells, cells attached to the surface of wells were transfected with DNA using TransIT-2020 or GeneXplus, at a DNA (μg) to TransIT-2020 or GeneXplus (μL) ratio of 1:3. Cells were harvested two days post-transfection.

### Western blot analysis

For denaturing gel conditions, cells were lysed in Laemmli sample buffer (120 mM Tris/HCl pH 6.8, 20% (v/v) glycerol, 4% SDS, 10% β-mercaptoethanol, 0.04% bromophenol blue) and boiled for 10 min at 100°C, separated by SDS-PAGE, transferred onto PVDF membranes. The membranes were blocked with 5% non-fat milk or 5% BSA in TBST for 0.5 h, incubated with primary antibodies at 4°C overnight or at room temperature for 2–3 h, followed by DyLight fluorochromes-conjugated or HRP-conjugated secondary antibodies. The membranes labeled with fluorescent secondary antibodies were visualized by direct infrared fluorescence detection with the Odyssey CLx (LI-COR). The membranes labeled with HRP-conjugated secondary antibodies were visualized with an ECL Prime Western Blotting System (Cytiva, RPN2232) using the Chemidoc Imaging System (Bio-Rad). For native gel conditions, cells were lysed in OverKleeft (OK) lysis buffer (50 mM Tris/HCl pH 7.5, 2 mM DTT, 5 mM MgCl_2_, 10% (v/v) Glycerol, 2 mM ATP, 0.05% (v/v) digitonin). Densitometric analysis on the immunoblots was performed using IMAGE STUDIO Lite software or Fiji (ImageJ).

### Immunoprecipitation

Cells from one 100-mm dish were lysed in 0.5 mL of lysis buffer (50 mM Tris pH 7.4, 150 mM NaCl, 0.5% (v/v) Nonidet P-40, 1% (v/v) Triton X-100, 5% (v/v) glycerol, 1% (v/v) phosphatase inhibitor cocktail 2 (Sigma-Aldrich, P5726), 1% (v/v) phosphatase inhibitor cocktail 3 (Sigma-Aldrich, P0044), protease inhibitor cocktail (Roche, 04693132001, ½ tablet for 10 mL lysis buffer)). Lysates were incubated on ice for 10 min, vortexed once or twice, and then isolated by centrifugation at 16 000× g for 10 min. Supernatants were transferred to a new tube and 50 μL of supernatants was kept as input. For immunoprecipitation of Flag-PSMC5 in HeLa cells, the remaining lysates were incubated with 20 μL of washed anti-Flag M2 magnetic beads (Merck, M8823) at 4°C for 3 h with gentle agitation. For immunoprecipitation of endogenous PSMC3 in BE(2)-M17 cells, the remaining lysates were incubated with 4 μL anti-PSMC3 antibody (Proteintech, 24142-1-AP) and incubated overnight at 4°C overnight with gentle agitation. Thereafter, 20 μL of washed Dynabeads protein A (Invitrogen, 10002D) was added to the samples and incubated at 4°C for 3 h with gentle agitation. Beads were washed three times with lysis buffer and proteins were eluted in 40 μL of Laemmli sample buffer, followed by boiling and analyzed by Western blot.

### FACS analysis of the degradation of Ub-G76V-GFP

BE(2)-M17 cells were transfected with the plasmid pUb-G76V-GFP-IRES-DsRed using TransIT-2020 or GeneXplus. After two days of transfection, cells were trypsinized and analyzed using an Attune NxT Flow Cytometer (ThermoFisher Scientific) using the BL1 (488 530/30) and YL1 (561 585/16) detectors. Cells were first gated on forward (FSC-A) and side scatter (SSC-A) for P1 and then for singlets (FSC-A/FSC-H) for P2. 20 000 single cells were recorded for each replicate. DsRed + gates were set using normal BE(2)-M17 cells. The ratio of BL1 (GFP) to YL1 (DsRed) signals in DsRed + cells was derived for each cell and the mean ratio was used for analysis. The data were analyzed using FlowJo software v10.7.1.

### Measurement of cell death with LDH cytotoxicity assay

Cell death in HeLa and SH-SY5Y cells after knockdowns of the 19S RP subunits was measured with the CyQUANT LDH cytotoxicity assay (Invitrogen, C20301), according to the manufacturer’s instructions. Briefly, after two days of siRNA knockdown (and drugs treatment) in 12-well plate or 24-well plate dishes, 50 μL of sample medium (every group contains an experimental LDH activity sample and a maximum LDH activity sample, and also a common cell-free medium LDH activity sample) was transferred to a 96-well flat-bottom plate in triplicate wells or duplicate wells. 50 μL of reaction buffer was added to each sample well and mixed. After incubating at room temperature for 30 min, 50 μL of stop buffer was added to each well and mixed. Absorbance was measured at 490 nm and 680 nm using a Tecan CM Spark plate reader. For each well, the 680-nm absorbance value (as a background) was subtracted from the 490-nm absorbance before calculation of cell death. The cell death was calculated by the following formula: cell death = (experimental LDH activity—cell-free medium LDH activity)/(maximum LDH activity—cell-free medium LDH activity), and then cell death of all the groups was normalized to untreated wild-type control or corresponding controls, as specified in the figure legends.

### Measurement of cell death with CellTox green cytotoxicity assay

Cell death in BE(2)-M17 cells was measured with the CellTox green cytotoxicity assay (Promega, G8743), according to the manufacturer’s instructions. Briefly, cells were plated onto 96-well plates and after cells attached to the bottom of the wells, medium was replaced with 100 μL of fresh medium containing CellTox green dye (at the dilution of 1:1000) and drugs. Cells were incubated in 5% CO_2_ at 37°C for two days and protected from light. Dead cells were imaged with the green channel and total cells were imaged using the phase channel using IncuCyte. The cell death was calculated by the following formula: cell death = green area of per well/phase area of per well, and then cell death of all the groups was normalized to untreated wild-type control or corresponding controls, as specified in the figure legends.

### Measurement of the activity and amount of proteasome complexes with in-gel proteasome assay

The in-gel proteasome assay was performed as previously described [32] with some modification.

Cells from one 100-mm dish were resuspended in 160 μL of OK lysis buffer (50 mM Tris/HCl pH 7.5, 2 mM DTT, 5 mM MgCl_2_, 10% (v/v) Glycerol, 2 mM ATP, 0.05% (v/v) digitonin), and kept on ice for 20 min with two or three times vortexes during this period. After centrifuging the samples at 4°C for 20 min at 20 000× g, supernatants were transferred to a new tube and protein concentration was determined using Pierce BCA protein assay kit (ThermoFisher Scientific, 23227) with bovine serum albumin as standard. 20 μg of native protein extracts with 1× NativePAGE Sample Buffer (ThermoFisher Scientific, BN2003) was loaded onto NuPAGE 3%–8%, Tris-Acetate gel (ThermoFisher Scientific, EA0375PK2 and EA03785BOX). Gels were run in 1× Novex Tris-Glycine Native Running Buffer (ThermoFisher Scientific, LC2672) with 0.5 mM ATP, 2 mM MgCl_2_, and 0.5 mM DTT at 160 V for 5 h at 4°C. For measuring in-gel proteasome activity, the gel was incubated in the reaction buffer (100 mM Tris/HCl pH 7.5, 1 mM ATP, 10 mM MgCl_2_, 1 mM DTT, 50 μM Suc-LLVY-AMC) for 30 min at 37°C in a dark box. The gel was scanned with UV Trans Illumination light with 530/28 filter using a ChemiDoc Imaging System (Bio-Rad). For measuring the amount of proteasome complexes, the native gel was soaked in solubilization buffer (2% SDS, 66 mM Na_2_CO_3_, 1.5% (v/v) β-mercaptoethanol) for 10–15 min at room temperature after measuring in-gel proteasome activity (if this was measured). Then the gel was transferred to PVDF membrane method in Tris-Glycine transfer buffer containing 20% (v/v) methanol and 0.04% SDS using wet electroblotting at a constant current of 60 mA at 4°C for 16 h. The membrane was blocked with 5% milk in TBST for 30 min and incubated with primary antibodies at 4°C overnight. After washing three times with TBST, the membrane was incubated with DyLight fluorochromes-conjugated secondary antibodies and scanned using Odyssey CLx (LI-COR). Densitometric analysis on the proteasome activity images and the immunoblots was performed using Fiji (ImageJ).

### Statistical analysis

All data are presented as mean values ± SD (standard deviation). Significance levels for comparisons between groups were determined using GraphPad Prism 9 (GraphPad Software) or Excel (Microsoft office). *P* values < 0.05 were considered statistically significant. For SDS-PAGE Western blots, protein levels were normalized to the house keeping protein GAPDH. Statistical analysis was performed using two-tailed, paired Student’s t-test or one-way ANOVA with post-hoc Dunnett’s multiple comparison test. The statistical parameters are specified in the figure legends.

## Supplementary Material

HMG-2024-CE-00118_YU_supplemental_data_ddae085
