# Supplementary material for: PSMC5 insufficiency and P320R mutation impair proteasome function
Source: Hum Mol Genet. 2024 May 22;33(17):1506–23. doi: 10.1093/hmg/ddae085 (PMC11336065; doi:10.1093/hmg/ddae085)
Supplement: HMG-2024-CE-00118_YU_supplemental_data_ddae085 [file hmg-2024-ce-00118_yu_supplemental_data_ddae085.pdf]

Supplementary data

PSMC5 insufficiency and P320R mutation impair proteasome function

Zhong-Qiu Yu, Jenny Carmichael, Galen Collins, Maria Daniela D'Agostino, Mathieu Lessard, Helen V. Firth, Pooja Harijan, Andrew E. Fry, John Dean, Jiuchun Zhang, Usha Kini, Alfred L. Goldberg, David C. Rubinsztein

TABLE OF CONTENTS

Figure S1 2
Figure S2 3
Figure S3 4
Figure S4 6
Figure S5 7

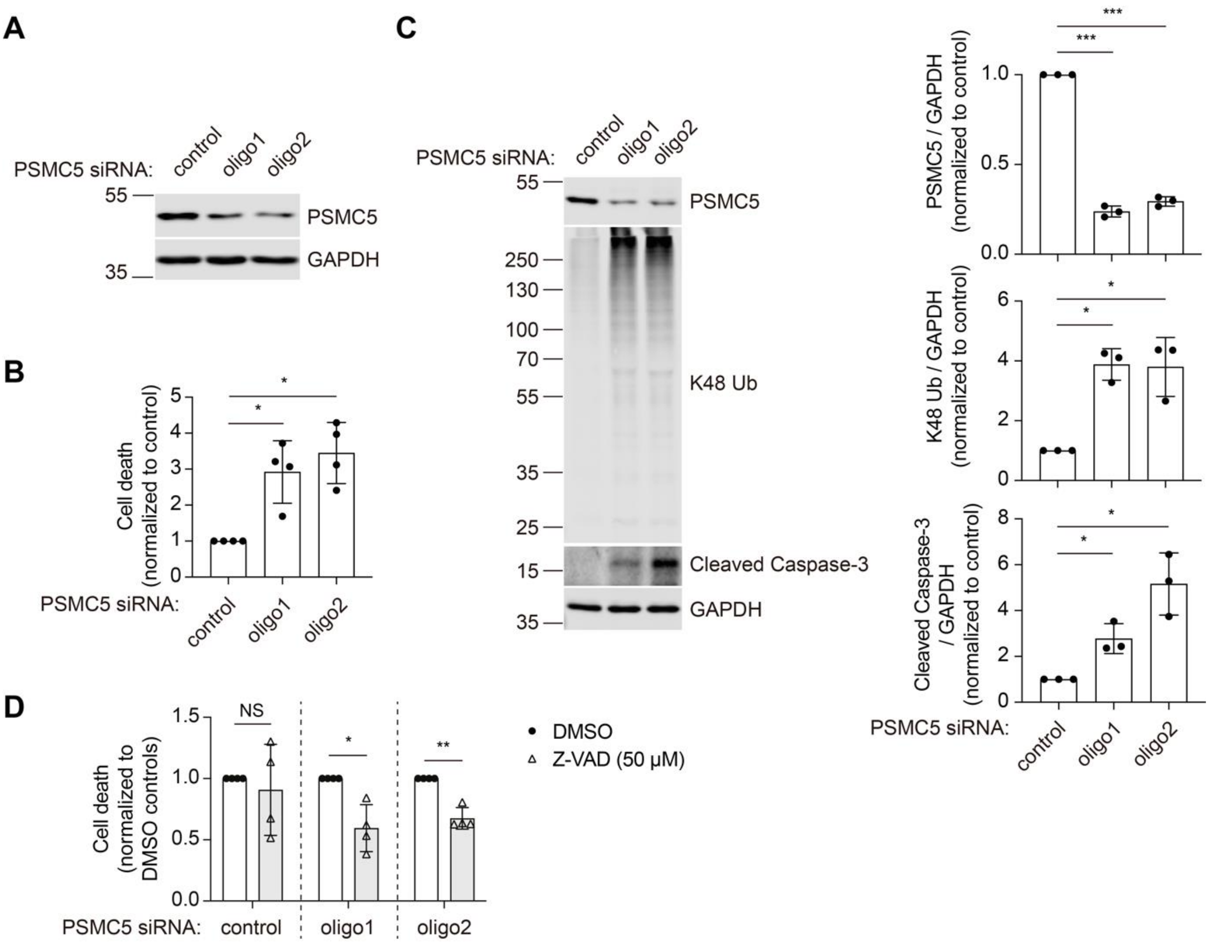

Figure S1. PSMC5 knockdown results in accumulation of K48-ubiquitinated proteins and apoptosis in SH-SY5Y cells. (A) Western blotting of PSMC5 levels after PSMC5 siRNA knockdown using oligo1 and oligo2. GAPDH served as protein loading control. (B) LDH cytotoxicity assay to measure cell death caused by PSMC5 knockdown. Plots represent mean ± SD (n = 4 independent experiments). P values were calculated using two-tailed, paired Student's t-test. (C) Western blotting and quantification of PSMC5, K48-ubiquitinated proteins, and cleaved caspase-3 levels in PSMC5-knockdown cells. Plots represent mean ± SD (n = 3 independent experiments). P values were calculated using two-tailed, paired Student's t-test. (D) LDH cytotoxicity assay to measure the inhibition effect of apoptosis inhibitor Z-VAD on cell death caused by PSMC5 knockdown. Cell death of Z-VAD treatment groups was normalized to DMSO treated wild-type, PSMC5 knockdown with oligo1 or oligo2, respectively. Plots represent mean ± SD (n = 4 independent experiments). P values were calculated using two-tailed, paired Student's t-test.

* indicates $P < 0.05$; ** indicates $P < 0.01$; *** indicates $P < 0.001$; NS, not significant.

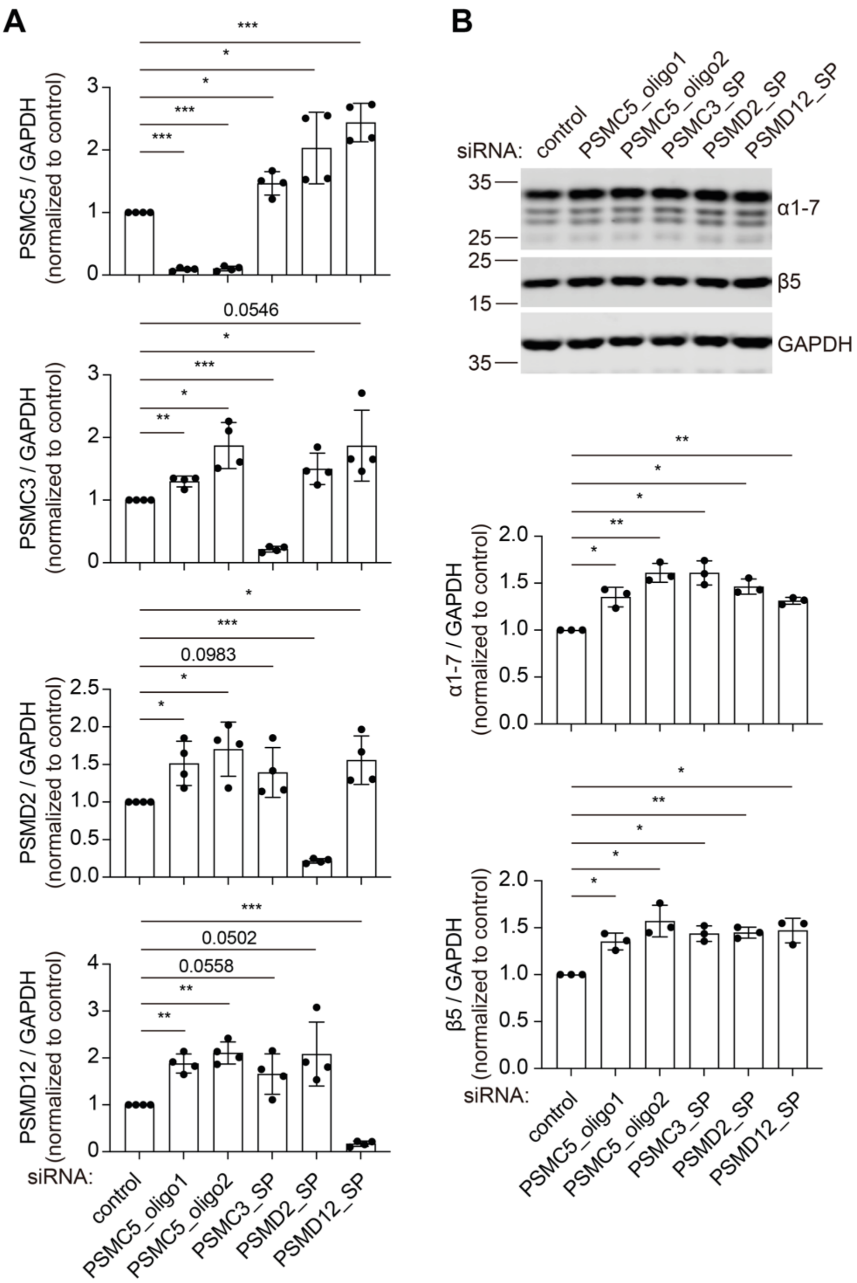

Figure S2. The protein levels of other subunits of proteasome increases upon knockdowns of the 19S RP subunits in HeLa cells. (A) Quantification of levels of PSMC5, PSMC3, PSMD2, and PSMD12 in PSMC5, PSMC3, PSMD2, or PSMD12-knockdown cells as representatively shown in Figure 2B. Plots represent mean ± SD (n = 4 independent experiments). P values were calculated using two-tailed, paired Student's t-test. (B) Western blotting of α1-7 and β5 and quantification thereof in PSMC5, PSMC3, PSMD2, or PSMD12-knockdown cells. GAPDH served as protein loading control. Plots represent mean ± SD (n = 3 independent experiments). P values were calculated using two-tailed, paired Student's t-test.

* indicates $P < 0.05$; ** indicates $P < 0.01$.

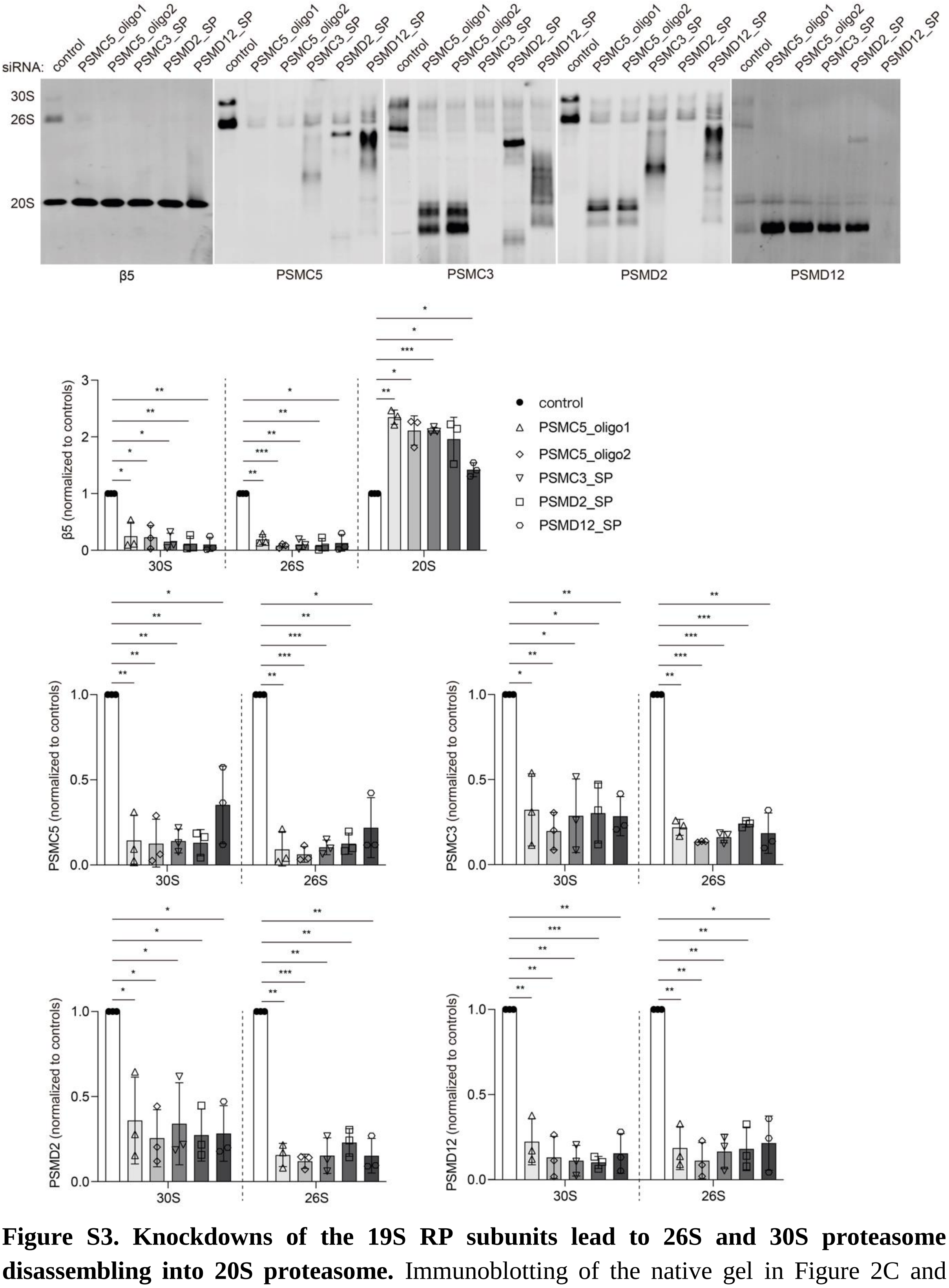

Figure S3. Knockdowns of the 19S RP subunits lead to 26S and 30S proteasome disassembling into 20S proteasome. Immunoblotting of the native gel in Figure 2C and probing of the membrane with anti-β5, anti-PSMC5, anti-PSMC3, anti-PSMD2, anti-PSMD12 antibodies and quantification of 30S, 26S, and 20S proteasome complexes indicated by β5, PSMC5, PSMC3, PSMD2, and PSMD12. The protein levels of β5, PSMC5, PSMC3, PSMD2, and PSMD12 in the 30S, 26S, and 20S proteasome complexes of different knockdowns were

normalized to the corresponding controls in the quantification plots. Plots represent mean ± SD (n = 3 independent experiments). P values were calculated using two-tailed, paired Student's t-test.

* indicates $P < 0.05$; ** indicates $P < 0.01$; *** indicates $P < 0.001$.

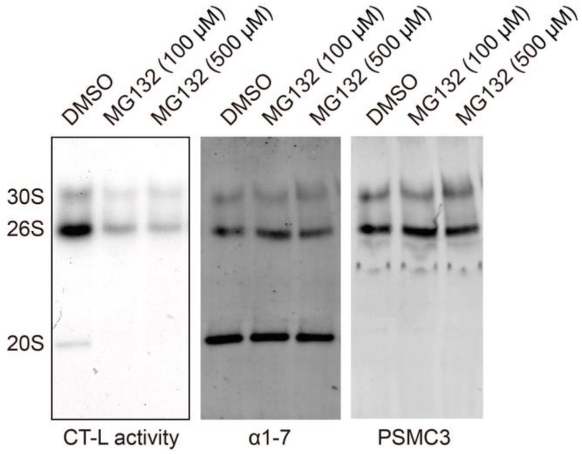

Figure S4. MG132 treatment inhibits proteasome activity measured using in-gel proteasome assay. In-gel proteasome CT-L activity of the 30S, 26S, and 20S proteasome complexes and immunoblotting of the native gel with anti-α1-7 and anti-PSMC3 antibodies. HeLa cell lysate (lysed by OK lysis buffer) was divided in three aliquots, each aliquot was incubated with DMSO, 100 μM MG132, or 500 μM MG132 for 15 mins on ice. After centrifuging, supernatant was loaded to native-gel for in-gel proteasome CT-L activity.

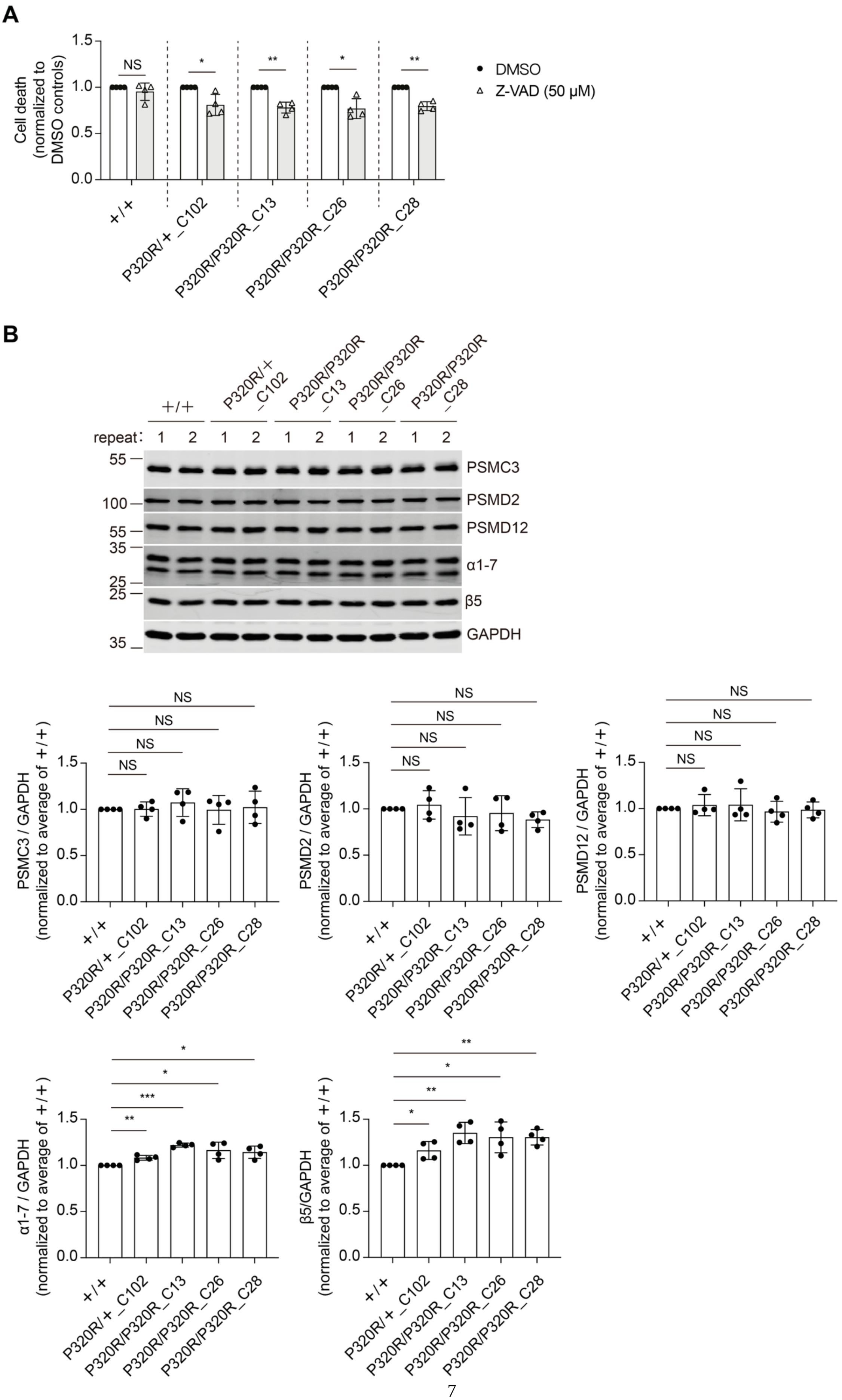

A
Cell death (normalized to DMSO controls)
DMSO
Z-VAD (50 μM)
NS
*
**
+/+
P320R/+ _C102
P320R/P320R_C13
P320R/P320R_C26
P320R/P320R_C28
B
repeat: 1 2 1 2 1 2 1 2 1 2
PSMC3
PSMD2
PSMD12
α1-7
β5
GAPDH
PSMC3 / GAPDH (normalized to average of +/+)
PSMD2 / GAPDH (normalized to average of +/+)
PSMD12 / GAPDH (normalized to average of +/+)
α1-7 / GAPDH (normalized to average of +/+)
β5/GAPDH (normalized to average of +/+)

Figure S5. The influence of PSMC5^{P320R} mutation on the protein levels of other subunits of proteasome. (A) CellTox green cytotoxicity assay to measure cell death of BE(2)-M17 cells carrying homozygous or heterozygous PSMC5^{P320R} treated with apoptosis inhibitor Z-VAD. Cell death of Z-VAD treatment groups was normalized to DMSO treated different cell lines, respectively. Plots represent mean ± SD (n = 4 independent experiments). P values were calculated using two-tailed, paired Student's t-test. (B) Western blotting and quantification of PSMC3, PSMD2, PSMD12, α1-7, and β5 in BE(2)-M17 cells carrying homozygous or heterozygous PSMC5^{P320R}. GAPDH served as protein loading control. The average of two repeats shown in one Western blot image was taken as a biological replicate. Plots represent mean ± SD (n = 4 independent experiments). P values were calculated using two-tailed, paired Student's t-test.

* indicates P < 0.05; ** indicates P < 0.01; *** indicates P < 0.001; NS, not significant. +/+: wild type; P320R/+: heterozygous PSMC5^{P320R}; P320R/P320R: homozygous PSMC5^{P320R}. C13, clone 13; C26, clone 26; C28, clone 28; C102, clone 102.
